# Exposure to Ambient Air Pollution and Cognitive Impairment in Community-Dwelling Older Adults: The Korean Frailty and Aging Cohort Study

**DOI:** 10.3390/ijerph16193767

**Published:** 2019-10-07

**Authors:** Jinyoung Shin, Seol-Heui Han, Jaekyung Choi

**Affiliations:** 1 Department of Family Medicine, Research Institute on Healthy aging, Konkuk University School of Medicine, Konkuk University Medical Center, Seoul 05030, Korea; jyshin@kuh.ac.kr; 2 Department of Neurology, Research Institute on Healthy aging, Konkuk University School of Medicine, Konkuk University Medical Center, Seoul 05030, Korea; alzdoc@kuh.ac.kr

**Keywords:** air pollution, aged, particulate matter, memory, cognition

## Abstract

The aim of this study was to investigate the associations between ambient air pollutants and cognitive impairment in Korean older adults. The cognitive function of 2,896 participants aged 70 to 84 years was measured using the Korean version of the mini-mental state examination, the digit span test, the word list learning test, and the frontal assessment battery. After matching the average concentrations of particulate matter (PM) <10 μm in size (PM_10_) and <2.5 μm (PM_2.5_), NO_2_, CO, SO_2_, and O_3_ between 2013 and 2017, the association between air pollutants and cognitive scales was analyzed using a linear mixed regression and a multiple logistic regression analysis (after adjusting for age, sex, health related behaviors, socioeconomic status, comorbidity, and meteorological data). Exposure to PM_2.5_, PM_10_, NO_2_, SO_2_, and CO was associated with cognitive impairment above and beyond age or education level effects. Specifically, PM_2.5_ was negatively associated with most components of the cognitive scales (interquartile range for PM_2.5_: 2.0 μg/m^3^, odds ratio for poor global cognition: 2.28, 95% confidence interval: 1.60–3.26). These associations may be affected by sex, residence area, or alcohol intake. Conclusively, air pollutants, especially PM_2.5_, were associated with cognitive impairment, including global cognition, attention, memory, and executive function in Korean older adults aged ≥70 years.

## 1. Introduction

Ambient air pollution, such as particulate matter (PM) <10 μm in size (PM_10_) and <2.5 μm (PM_2.5_), nitrogen dioxide (NO_2_), carbon monoxide (CO), sulfur dioxide (SO_2_), and ozone (O_3_), has recently been recognized as one of the most serious health issues worldwide. A growing body of research has reported that air pollution is associated with impaired cognitive function and neuropathology reminiscent of neurodegenerative disorders, including dementia, Alzheimer’s disease, and Parkinson’s disease [1,2,3]. Cognitive impairment is a growing health problem for older adults. Up to 42% of older adults worldwide have been reported to have mild cognitive impairment [4]. Patients with mild cognitive impairment have been shown to be at risk of developing Alzheimer’s disease and other neurodegenerative diseases [5,6]. The global number of individuals who live with dementia has increased rapidly, mainly due to the increase in the aging population and population growth, in general [7]. Multiple risk factors have been associated with the development and worsening of cognitive impairment, such as cardiovascular disease, reduced testosterone, insulin resistance, inflammation, health-related behaviors, and mental health status [4,8,9].

Although several risk factors for cognitive impairment are known, the causal links between ambient air pollution and cognitive impairment are not clear. In particular, previous studies have focused on the association between particulate matter or ozone and neurodegenerative disorders [10,11]. Due to the fact that PM occurs secondarily as a result of complex photochemical reactions with NOx or SOx [12], we have to consider the associations between NO_2_, SO_2_, and neurodegenerative disorders. Nevertheless, evidence for the associations between CO, SO_2_, and NO_2_ and neurodegenerative disorders is lacking. Many previous studies on the relationship between air pollution and a decline in cognitive abilities investigated one main outcome, such as the incidence of dementia, Parkinson’s disease, or hospitalization due to Alzheimer’s disease, which had already progressed to neurodegenerative disease [13,14,15]. Furthermore, in previous studies, the assessment tool for cognitive function was often limited to working memory or the mini-mental state examination (MMSE) [1,11,16]. The aforementioned large study on the effect of air pollution in individuals with mild cognitive impairment identified Alzheimer’s disease and dementia [2], but it was carried out in Western populations. Therefore, differences in race, lifestyle, air-pollutant concentration, and exposure duration may have affected the results. The level of air pollution in Korea has been shown to be higher than in the UK or the US (two countries in which the associations between cognitive impairment and air pollution have been shown) [2,17,18]. However, there has been little research on this association for the Korean older population.

This study aimed to investigate the association between ambient air pollutants and cognitive impairment, measured by a comprehensive assessment tool in Korean older adults, including the effect of health-related behavior and socioeconomic status.

## 2. Materials and Methods

### 2.1. Study Participants

The Korean Frailty and Aging Cohort study (KFACS) is an ongoing multicenter longitudinal study initiated in 2016. Sex- and age-stratified community residents aged 70 to 84 years were recruited at 10 centers in urban and rural regions throughout South Korea. From the total 3014 adults enrolled in 2016–2017, we analyzed 2896 subjects (96.1%) after excluding the following residents: those that had moved within one-year (n = 99), those that had a diagnosis of dementia by a physician (n = 8), and those for whom we were missing residence information (n = 11). The protocol of the Korean Frailty and Aging Cohort study was approved by the Institutional Review Board (IRB) of the Clinical Research Ethics Committee of Konkuk University Medical Center, Seoul, Korea, and all participants provided written informed consent (IRB File Number: KUH1230027).

### 2.2. Air Pollutant Variables

We obtained the average concentrations of PM_10_, NO_2_, CO, SO_2_, and O_3_, as measured hourly by the Korean Air Pollutants Emission Service in 2013–2017. Due to the fact that PM_2.5_ has only been measured in Korea since 2015, we applied the average concentrations of PM_2.5_ in the data from 2015 to 2017. The level of air pollutants measured at 268 nationwide surveillance stations located in residential areas was matched with the location of the participants’ surveillance centers, and the average concentrations were calculated.

PM_10_ and PM_2.5_ were measured using β-ray attenuation (PM–711D, DONGIL GREENSYS, Seoul, Korea). NO_2_ was measured using chemiluminescence (CM2041, APM ENGINEERING CO., LTD, Gyeonggi-do, Korea). CO was measured using a nondispersive infrared sensor (ZKJ, DONGIL GREENSYS, Seoul, Korea). SO_2_ was measured with UV fluorescence (CM2050, APM ENGINEERING CO., LTD, Gyeonggi-do, Korea). O_3_ was measured using ultraviolet photometry (202, TOTAL ENGINEERING CO., LTD, Gyeonggi-do, Korea). All measurements of air pollutants were decided according to the Wireless Distributed Environmental Sensor Networks, which are stable to diverse environmental conditions [19]. The air pollutant measurements were made according to the standard operating procedure of the Korean Air Pollutants Emission Service of the National Institute of Environmental Research (Incheon, South Korea). The level of air pollutants and meteorological data, including temperature, rainfall, and wind speed are presented in Supplementary Table S1.

### 2.3. Cognitive Impairment

Cognitive impairment was assessed using the Korean version of the Consortium to Establish a Registry for Alzheimer’s Disease Assessment Packet (CERAD-K) including the Korean version of the MMSE (MMSE-KC), the digit span test, the word list learning test [20,21], and the frontal assessment battery (FAB) [22]. The MMSE is one of the most frequently used cognitive screening tools measuring orientation, memory, attention/concentration, language, and visuospatial function [23]. The Korean normative study of the MMSE was administered. Scores of 19 or less indicated severe cognitive impairment, scores of 20–23 indicated mild cognitive impairment, and scores of 24 or greater represented normal ability [24]. The original scale yielded a reliability of 0.92 [23]. The digit span test assessed concentration, attention, and immediate memory by asking participants to repeat a string of numbers in forward and backward order. The total score represented the longest series repeated without error [25]. The word list learning test (0–30 points) consisted of memory, recall, and word list recognition, in which the subjects were asked to memorize 10 visually presented words and the ability to learn new information was measured. In the word list recall test (0–10 points), the participants were asked to recall the words presented in the word list learning test after a delay, and their word recall ability was measured. In the word list recognition test (0–10 points), the participants were asked to distinguish the words presented in the word list learning test from new words, and their visual word recognition ability was measured [26]. FAB has become the most widely used screening tool for executive function. It takes a short time to complete and is easy to administer in clinical settings [27]. Executive function is defined as a variety of high-level cognitive abilities, such as planning, working memory, mental flexibility, and inhibition [28]. The FAB consisted of six subtests, which assessed conceptualization, mental flexibility, motor programming, sensitivity to interference, inhibitory control, and environmental autonomy. FAB scores were calculated from the summation of all subtests. The test has been used to identify Alzheimer’s disease and other types of dementia, as well as psychiatric diseases and neurological problems, including movement disorders [28]. Higher values indicate better executive function (range 0–18). All assessments were administered by trained researchers and the data obtained were audited by a research modulator.

### 2.4. Other Variables

Questionnaires concerning socioeconomic status, the Korean version of activities of daily living instrument (K-ADL), the Korean version of instrumental activities of daily living (K-IADL), medical histories, and health-related behaviors were surveyed by trained researchers. We categorized smoking status as being either current (for residents who were smoking daily or intermittently at the time of the survey), or never/former (for residents who smoked in the past but were not currently smoking). To account for alcohol consumption, we categorized participants as either “never”, “less than once per week”, or “more than once per week”. Physical inactivity was defined as the lowest 20% of the sex-specific total energy consumed (kcal/week), which was assigned by the intensity of physical activity following metabolic equivalents (MET = kcal kg^−1^ h^−1^) using the International Physical Activity Questionnaire. Participants with less than 494.65 kcal/week for men, or less than 283.50 kcal/week for women, were defined as being physically inactive [29].

We also obtained the following demographic information: years of education (<9, ≥9), marital status (married/with partner and divorced/widowed/unmarried), household income (<1,000,000 won/month or ≥1,000,000 won/month), length at current residence (1 to 5 years/>5 years), and place of residence (rural or urban). Body mass index (BMI) was calculated as body weight divided by height squared (kg/m^2^). Carlson’s comorbidity index was used to indicate overall health status, and was calculated using 18 conditions, excluding dementia [30].

### 2.5. Statistical Analyses

Comparison of continuous variables (i.e., demographics, socioeconomic characteristics, and health-related behaviors of the study population) are presented as means with standard errors. Categorical variables are presented as numbers and percentages. As the level of air pollutants and cognitive scores were not normally distributed according to the Shapiro–Wilk test, we evaluated the relationship between the average annual concentrations of air pollutants and the cognitive scales using Spearman’s correlation analysis (Supplementary Table S2). We used log-transformed cognitive scales in the following analysis because of the non-normal distribution. The *β* coefficients (95% confidence intervals (CI)) for the log-transformed cognitive scales per one standard deviation (SD) increase in air pollutants were analyzed by a linear mixed model. In all analyses, each center was adjusted as a random effect. Age, sex, body mass index, smoking, alcohol intake, physical activity, education, household income, marital status, Carlson’s comorbidity index, length of time at the same residence, meteorological data, residence area, PM_2.5_, PM_10_, CO, SO_2_, NO_2_, and O_3_ were adjusted as fixed effects [25]. We evaluated the odds ratios for the lowest quartile of log-transformed cognitive scales according to the per interquartile increase in PM_2.5_, PM_10_, NO_2_, SO_2_, CO, and O_3_ using multiple logistic regression analysis (after adjusting for confounding factors). We conducted stratified analyses to investigate possible effect modifications by demographics, socioeconomic characteristics, and health-related behaviors in subgroup analysis. Two-tailed *P* < 0.05 was considered significant. All statistical analyses were conducted using IBM SPSS for Windows, version 24.0 (IBM Corp., Armonk, NY, USA).

## 3. Results

### 3.1. Demographic Characteristics of the Study Population

The demographics, socioeconomic characteristics, and health-related behaviors of the study population are summarized in Table 1. The mean age was 76 years (range 70–84) and women represented 52.5% of the participants. Among the total participants, 77.8% had lived more than five years in their current residence and about 70% resided in urban areas. (Table 1).

### 3.2. Cognitive Scales according to the Increases in Air Pollutants

#### 3.2.1. Linear Mixed Model

The variations in cognitive scales per increase of one standard deviation in the air pollutants, after adjusting for all covariates, including meteorological data, are presented in Table 2. PM_2.5_ was negatively associated with all cognitive scales. The magnitude of the coefficients for PM_2.5_ was larger than the decrease in cognitive function for each increase of one year in age. PM_10_ was negatively associated with the MMSE-KC scores and the digit span-forward scores. PM_10_ showed null or positive associations with memory scales. CO, SO_2_, and NO_2_ showed negative associations with memory scales, however, there were inconsistent findings between CO, SO_2_, NO_2_, O_3_, and cognitive scales.

#### 3.2.2. Multiple logistic model

**Figure 1:**
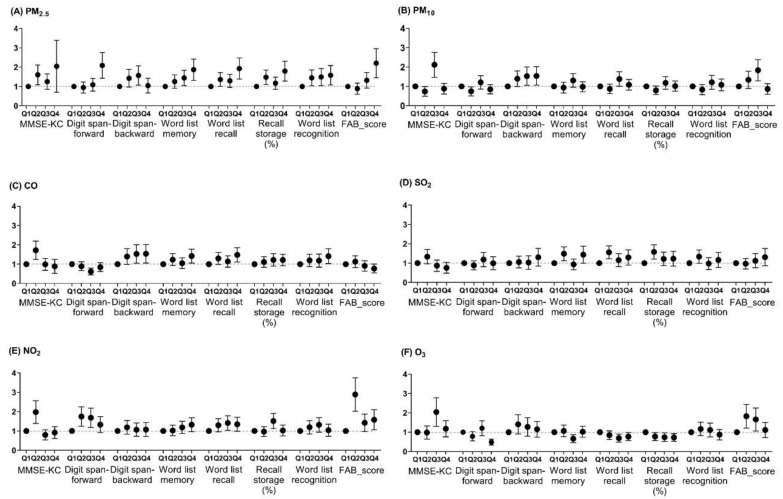
Odds ratios and 95% confidence intervals for the lowest quartile of log-transformed cognitive scales according to quartiles of air pollutants.

Odds ratios for the lowest quartile of log-transformed cognitive scales according to the per interquartile increase of air pollutants are presented in Figure 1. An increase in PM_2.5_ quartile level was associated with an increased probability of the low cognitive scales, including global cognition, attention, memory, and executive function (all *P* < 0.05). PM_10_, NO_2_, and O_3_ were associated with higher risks for poor global cognition and executive function. CO was associated with a higher risk for poor global cognition, low attention, and executive function. SO_2_ was associated with a higher risk for poor global cognition and memory function. Except for PM_2.5_, these associations tended to be inconsistent.

### 3.3. Cognitive Impairment Due to the Increase of PM_2.5_ and Participants’ Characteristics

**Figure 2:**
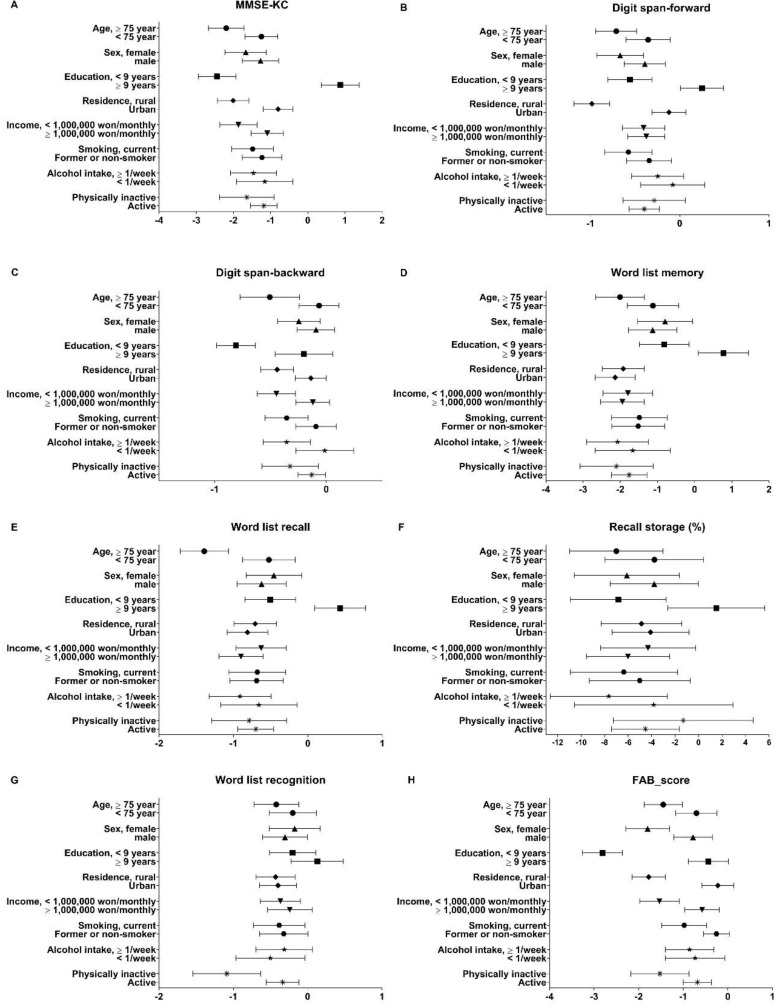
The *β* coefficients of cognitive scales according to the increase of particulate matter (PM) <2.5 μm (PM_2.5_) according to participants’ characteristics.

We found negative associations between PM_2.5_ and the following attributes: increased age, female sex, less than nine years of education, rural residence, low income, current smoking, alcohol intake more than once per week, and physical inactivity (Figure 2). However, it varied according to the cognitive scales. In the subgroup analysis, we confirmed the effect modification for sex, residence area, or alcohol intake (*P*-value > 0.05). In female participants, recall storage scores showed a negative association with PM_2.5_, while in male participants, they did not. Being male showed a negative association between the recognition test and PM_2.5_. However, females had a null association with this test. Rural residents showed negative associations with PM_2.5_ and digit span test and FAB score, while participants living in urban areas had null associations. Alcohol intake of more than once per week was associated with cognitive impairment related to PM_2.5._ Alcohol intake of less than once per week was associated with lower scores in the recognition test. Participants with education level more than nine years showed the null associations with increase of PM_2.5._

## 4. Discussion

In the present cross-sectional study on Korean older adults, we evaluated the associations between air pollutants and cognitive function, including global cognition, attention, memory, and executive function. We found that exposure to PM_2.5_, PM_10_, NO_2_, SO_2_, and CO was associated with cognitive impairment. We found a strong association between higher PM_2.5_ exposure and worse performance in most of the cognitive scales. The effect of air pollutants on the cognitive scales was significant, compared to that of age and education level. Specifically, global cognition, attention, and recall memory were vulnerable to exposure to air pollutants. Most of the existing evidence for a link between air pollution and cognitive impairment comes from studies of patients diagnosed with dementia [7,10,13,14], or of populations younger than our study’s participants (mean age: 56.3 years vs. 76.0 years) [11]. This study adds to existing research on air pollution and cognition in older adults by demonstrating links between various pollutants, such as NO_2_, SO_2_, and CO, and the scores on various cognitive scales for older Korean adults (age ≥ 70), considering all possible confounding factors.

However, we did not find a linear association between air pollutants and cognitive function, as in a previous study [16]. We believe that the reason for this finding is a threshold effect at low levels of air pollutants [31]. Below a cut-off value for the air pollutants, the effect may be not clear. Therefore, we may find out the difference associations in Table 2 and Figure 1. We also found several effect modifications for sex, residence area, and alcohol intake. Females were vulnerable in terms of recall storage and males were vulnerable in terms of recognition. The prevalence of Alzheimer’s disease and other dementias have a higher prevalence in females than in males [7]. However, the association between air pollution and cognitive impairment should be considered not only with respect to the longer lifespan of females [7], but also the higher exposure from transport use [32] or proven risks in men [31]. Therefore, a mixed pattern may be represented with respect to sex. On the other hand, participants residing in rural areas showed worse cognitive function in our study. A possible cause could be the indoor pollution in rural areas, although we did not measure the level of indoor pollution [33]. The air purifier market has tripled from 2016 to 2018 in Korea [34]. The use of air purifiers is expected to have an impact on the association between air pollution and cognition in the future.

In this study, among the air pollutants, PM_2.5_ was confirmed as an obvious risk factor for cognitive impairment, supporting several previous studies [35]. The adverse neurological mechanism of air pollutants may be related to neuro-inflammation associated with oxidative stress and cytokine production [36,37]. Exposure to high ambient PM_2.5_ accelerated cognitive decline and all-cause dementia up to 21% in US community-dwelling older adults aged 65 to 79 years [38]. Systemic exposure to PM_2.5_ potentially contributes to the inflammatory, glial, and amyloid pathology responses via bloodstream uptake, and to direct infiltration of the central nervous system [1,38]. However, in our study, the associations between PM_10_, NO_2_, SO_2_, CO, O_3_, and the cognitive scales were not evident. There are three possible reasons for this. First, the effect of PM_2.5_ on cognitive impairment may be remarkable compared to other pollutants. The contributions of soil dust and natural sources of PM_2.5_ were smaller than they were for PM_10_ in Korea [39], while a high contribution of PM_2.5_, which comes from secondary aerosol sources via the atmospheric chemical reaction of gaseous pollutants, such as SO_2_ and NO_2_ that originate from fuel combustion, could be more hazardous to human health [39]. Second, very small and soluble PM_2.5_ can cross the blood–brain barrier, accessing the central nervous system and contributing to the development of neurotoxicity, unlike PM_10_ [3]. It has been suggested that the reduced systemic effects of PM_10_ result from the inability of coarse fraction PM to infiltrate the bloodstream or directly assess the brain. Third, an area-based approach, which assigns exposures at the level of each participant’s community, county, postcode, or census tract, may be more adequate for evaluating the effects of PM_2.5_, which is dispersed relatively homogenously across space, than for PM_10_, NO_2_, SO_2_, CO, and O_3_ [35]. This potential measurement error, or exposure estimation error, may provide a possible explanation for the heterogeneity observed in previous studies on cognitive impairment for different pollutants, statistical models, geographic regions, and time periods [33,35]. In Mexican older adults aged ≥60 years with five-year exposures at a mean PM_2.5_ concentration of 12.9 μg/m^3^ (min–max 2.4–27.8 μg/m^3^), PM_2.5_ was negatively associated with cognitive function, as in our study (mean: 25.5 μg/m^3^, min–max 23.4–28.5 μg/m^3^) [33], although the study examined longer exposure durations and lower levels than our study.

We did not find any link between O_3_ and cognitive impairment in our study. Moreover, the positive associations between O_3_ and MMSE-KC scores, digit-forward span scores, word list recall, recall storage, and FAB scores were unexpected results, compared to a previous limited study [10]. However, a positive association between O_3_ and the logical memory of adults aged 60 and older was found in a study by Gatto et al. in California, USA (2000–2006) [40]. Interestingly, a positive association was seen at the range of 34–49 ppb in O_3_, which was similar to the level in our study (21.6–36.5 ppb), while the null association was seen at a higher level in that study (>49 ppb in O_3_) [40]. In a Taiwanese study by Jung et al., 10-year exposure to O_3_ in people aged ≥ 65 years increased the risk of Alzheimer’s disease, according to the International Classification of Diseases, ninth revision [10]. The level of O_3_ in the study by Jung et al. was higher than in our study (mean 88.97 ± 7.80 ppb and range 52.79–106.74 ppb in Jung et al. vs. mean 26.3 ± 4.3 ppb and range 21.6–36.5 in our study). Although we can suggest that the discrepancies were due to different levels of O_3_ at different latitudes or exposure of the participants, further research is needed to explore the association between O_3_ and cognitive function.

This study had several limitations. First, it was not possible to establish causality because this was a cross-sectional study based on a recruitment survey from a cohort study. Second, we matched the community of residence and local air pollutant levels using the enrollment center location. Therefore, measurement errors for the air pollutants are possible. Third, undiagnosed dementia patients with low MMSE-KC scores may have been included in this study, although we initially excluded patients diagnosed by a physician with dementia. Fourth, due to the lack of information about the exposure window related to the development of an individual’s cognitive impairment, we could only estimate the air pollutant exposure duration from the data in previous studies [1,2,16]. Excluding participants (n = 99) who moved within one year considering the minimum exposure or the difference in exposure duration for PM_2.5_ (2015–2017) and non-PM_2.5_ (2013–2017) may have biased study findings. Finally, genetic predisposition (such as having the *APOE-4* gene, which may increase the risk of Alzheimer’s disease), and traffic-related exposure were not considered in our models. However, we made an effort to minimize enrollment selection bias by matching age and sex for the older adults, and by obtaining missing values by visiting participants’ homes or by surveying via telephone. We also performed our analysis after adjustment for several confounding variables.

## 5. Conclusions

Air pollutants, especially PM_2.5_, were associated with cognitive impairment, including global cognition, attention, memory, and executive function in Korean older adults aged ≥70 years. These associations differed according to sex, residence area, and health-related behavior, such as alcohol intake. This information may be helpful for policy-making to control air pollution as a risk factor for cognitive impairment.

## Figures and Tables

**Table 1:** Characteristics of this study populations (n = 2896).

Variables	Mean ± SD or N (%)
Age, (min/max), years	76.0 ± 3.9 (70.0/84.0)
Sex	
Male	1377 (47.5)
Female	1519 (52.5)
Smoking	
Current	1104 (38.1)
Never/former	1792 (61.9)
Alcohol intake	
Never/Less than one time per week	520 (18.0)
More than one time per week	2376 (82.0)
Physical activity, kcal/week	
Active	2580 (89.1)
Inactive	316 (10.9)
Education, years	
<9	1396 (48.2)
≥9	1500 (51.8)
Marital status	
Married/with partner	1950 (67.3)
Divorced/widowed/unmarried	946 (32.7)
Household income, won/monthly	
<1,000,000	1370 (47.3)
≥1,000,000	1526 (52.7)
Length of current residence, year	
1~5	643 (22.2)
>5	2253 (77.8)
Residence	
Urban	2013 (69.9)
Rural	883 (30.1)
Body mass index, kg/m^2^	24.5 ± 3.1
K-ADL (min/max)	7.1 ± 0.4 (7/14)
K-IADL (min/max)	0.3 ± 0.8 (0/9)
Carlson comorbidity index (range: 0–7)	0.48 ± 0.79
MMSE-KC (range: 8–30)	25.6 ± 3.3
Digit span-forward (range: 0–9)	5.8 ± 1.5
Digit span-backward (range: 0–8)	3.3 ± 1.1
Word list memory (range: 0–29)	16.7 ± 4.3
Word list recall (range: 0–10)	5.5 ± 2.1
Recall storage (%)	77.4 ± 24.2
Word list recognition, (range: 0–10)	8.6 ± 1.9
Frontal assessment battery test (range: 0–18)	13.4 ± 3.0

K-ADL: Korean version of activities of daily living instrument, K-IADL: Korean version of instrumental activities of daily living instrument, MMSE-KC: Korean version of the mini-mental state examination. Physical inactivity was defined as the lowest 20% of the gender-specific total energy consumed (kcal/week); <494.65 kcal for men or <283.50 kcal for women.

**Table 2:** Results of regression models ^1^ for cognitive scales according to the increases of air pollutants and participants’ characteristics.

	1-SD	MMSE-KC	Digit Forward Span	Digit Backward Span	Word List Memory	Word List Recall	Recall Storage	Word List Recognition	FAB_Score
PM_2.5_	1.5 μg/m^3^	−0.010 (−0.019,−0.001)^2^	−0.022 (−0.040,−0.005)^2^	−0.039 (−0.057,−0.020)^2^	−0.024 (−0.036,−0.011)^2^	−0.036 (−0.054,−0.018)^2^	−0.024 (−0.038,−0.010)^2^	−0.016 (−0.029,−0.003)^2^	−0.037 (−0.048,−0.025)^2^
PM_10_	4.6 μg/m^3^	−0.035 (−0.050,−0.020)^2^	−0.029 (−0.057,−0.001)^2^	0.022 (−0.008,0.053)	0.049 (0.015,0.083)^2^	−0.011 (−0.033,0.011)	−0.014 (−0.031,0.003)	0.001 (−0.015,0.016)	−0.002 (−0.021,0.017)
CO	0.08 ppm	0.044 (0.032,0.056)^2^	0.052 (0.029,0.075)^2^	−0.022 (00.046,0.003)	−0.035 (−0.063,−0.007)^2^	−0.018 (−0.034,−0.003)^2^	−0.005 (−0.017,0.007)	−0.007 (−0.018,0.004)	−0.006 (−0.019,0.007)
SO_2_	0.9 ppb	0.007 (−0.005,0.020)	0.058 (0.035,0.082)^2^	−0.032 (−0.058,−0.007)^2^	−0.010 (−0.039,0.019)	−0.038 (−0.060,−0.017)^2^	−0.022 (−0.039,−0.006)^2^	−0.011 (−0.026,0.004)	0.007 (−0.005,0.020)
NO_2_	7.7 ppb	0.012 (0.001,0.025)^2^	−0.026 (−0.050,−0.003)^2^	0.015 (−0.009,0.040)	0.034 (0.006,0.063)^2^	−0.019 (−0.035,−0.003)^2^	−0.007 (−0.042,0.015)	−0.003 (−0.015,0.008)	−0.019 (−0.034,−0.005)^2^
O_3_	4.3 ppb	0.045 (0.027,0.062)^2^	0.062 (0.029,0.094)^2^	−0.029 (−0.064,0.006)	0.009 (−0.031,0.048)	0.034 (0.018,0.049) ^2^	0.013 (0.001,0.025)^2^	0.010 (−0.001,0.021)	0.011 (0.002,0.019)^2^
Age	1 year	−0.006 (−0.008,−0.005)^2^	−0.009 (−0.012,−0.006)^2^	−0.009 (−0.012, −0.006)^2^	−0.020 (−0.024,−0.017)^2^	−0.026 (−0.030,−0.022)^2^	−0.012 (−0.016,−0.009)^2^	−0.010 (−0.013,−0.007)^2^	−0.009 (−0.011,−0.007)^2^
Education	1 year	−0.011 (−0.010,−0.012)^2^	−0.018 (−0.016,−0.020)^2^	−0.024 (−0.021,−0.026)^2^	−0.016 (−0.014,−0.019)^2^	−0.017 (−0.013,−0.021)^2^	−0.005 (−0.002,−0.008)^2^	−0.006 (−0.003,−0.009)^2^	−0.023 (−0.021,−0.025)^2^

^1^ β coefficients (95% confidence intervals) of log-transformed cognitive scales per one standard deviation increase of air pollutants or one year of age (increase) and education (decrease) were assessed by linear mixed model. Age, sex, body mass index, smoking, alcohol intake, physical activity, education, household income, marital status, Carlson’s comorbidity index, length of same residence, meteorological data, residence area, PM_2.5_, PM_10_, CO, SO_2_, NO_2_, O_3_ (selected air pollutant was not included in the model) were adjusted as fixed effects, and each center was adjusted as random effects. Although there were highly correlated among the air pollutants, we confirmed the unchanged overall results when excluding combinations of these variables (data were not shown). 2P < 0.05, SD: standard deviation, PM_2.5_: particulate matter <2.5 μm in diameter, PM_10_: particulate matter <10 μm in diameter, NO_2_: nitrogen dioxide, SO_2_: sulfur dioxide, CO: carbon monoxide, O_3_: ozone. MMSE-KC: Korean version of the mini-mental state examination; FAB: frontal assessment battery test score.

## Supplementary Materials

Supplementary materials are available online at https://www.mdpi.com/1660-4601/16/19/3767/s1. Table S1: Distribution of annual concentrations of air pollutants and meteorological data in 2013–2017, Table S2: Correlations between cognitive scales and the annual concentrations of air pollutants, 2013–2017^*^ (PM_2.5_: 2015–2017).
