# Supplementary material for: Exposure to Ambient Air Pollution and Cognitive Impairment in Community-Dwelling Older Adults: The Korean Frailty and Aging Cohort Study
Source: Int J Environ Res Public Health. 2019 Oct 7;16(19):3767. doi: 10.3390/ijerph16193767 (PMC6801547; doi:10.3390/ijerph16193767)
Supplement: Supplementary file 1 [file ijerph-16-03767-s001.pdf]

International Journal of
Environmental Research and Public Health

MDPI

Table S1. Distribution of annual concentrations of air pollutants and meteorological data in 2013-2017.

Variables	Mean	SD	IQR	Percentiles				
				Minimum	25th	50th	75th	Maximum
Air pollution								
$PM_{2.5}$, μg/m^3	25.5	1.5	2.0	23.4	24.5	25.1	26.5	28.5
PM_{10}, μg/m^3	47.1	4.6	5.4	37.2	44.4	47.0	49.8	61.4
CO, 10 ppm	5.3	0.8	1.2	3.6	5.0	5.1	6.2	6.4
SO_2, ppb	4.5	0.9	1.4	3.0	3.9	4.9	5.3	5.7
NO_2, ppb	23.6	7.7	13.5	10.0	18.1	25.6	31.6	34.5
O_3, ppb	26.3	4.3	5.2	21.6	22.6	23.9	28.7	36.5
Weather conditions								
Temperature, °C	13.2	10.3	20.4	-3.4	2.7	14.8	23.1	28.0
Rainfall, mm	87.2	126.2	63.3	1.0	23.2	47.2	86.5	676.2
Wind speed, m/s	2.5	0.37	0.6	1.8	2.2	2.4	2.8	3.4

SD: standard deviation, IQR: interquartile range, $PM_{2.5}$: particulate matter < 2.5 μm in diameter, PM_{10}: particulate matter < 10 μm in diameter, NO_2: nitrogen dioxide, SO_2: sulfur dioxide, CO: carbon monoxide, O_3: ozone. The concentration of $PM_{2.5}$ was measured from 2015 to 2017. Mean levels of temperature, rainfall and wind speed per monthly were shown at Seoul (Lat. (N) 37°34′, Long. (E) 126°57′) in 2013-2017. http://www.weather.go.kr/weather/climate/average_world_monthly.jsp.

Table S2. Correlations between cognitive scales and the annual concentrations of air pollutants, 2013-2017 ($PM_{2.5}$: 2015-2017).

Air Pollutants	Global Cognition	Attention		Memory				Executive Function	$PM_{2.5}$	PM_{10}	CO	SO_2	NO_2	O_3
	MMSE-KC	Digit Span -Forward	Digit Span -Backward	Word List Memory	Word List Recall	Recall Storage (%)	Word List Recognition	FAB_ Score						
$PM_{2.5}$	-0.16[1]	-0.14[1]	-0.13[1]	-0.161	-0.141	-0.09[1]	-0.10[1]	-0.16[1]	1.00	0.49[1]	0.38[1]	0.10[3]	0.10[1]	-0.19[1]
PM_{10}	0.02	0.01	0.06[2]	-0.07[1]	-0.05[3]	-0.03	-0.05[3]	0.02		1.00	0.68[1]	0.19[1]	0.22[1]	-0.28[1]
CO	0.08[1]	0.01	0.06[3]	-0.04	-0.03	-0.02	-0.06[2]	0.07[1]			1.00	0.49[1]	0.47[1]	-0.59[1]
SO_2	0.08[1]	0.06[2]	0.07[1]	0.08[1]	0.02	-0.04[3]	0.01	0.04[3]				1.00	0.84[1]	-0.71[1]
NO_2	0.07[1]	-0.01	0.11[1]	0.07[1]	0.01	-0.02	-0.01	0.05[3]					1.00	-0.90[1]
O_3	-0.10[1]	0.02	-0.17[1]	-0.08[2]	-0.03	0.02	-0.01	-0.12[1]						1.00

[1]$P < 0.05$, [2]$P < 0.01$, [3]$P < 0.05$.Spearman's correlation analysis was used, because of non-nominal distribution. $PM_{2.5}$: particulate matter < 2.5 μm in diameter, PM_{10}: particulate matter < 10μm in diameter, NO_2: nitrogen dioxide, SO_2: sulfur dioxide, CO: carbon monoxide, O_3: ozone. MMSE-KC: Korean version of the Mini-Mental State Examination; FAB_score: total Frontal assessment battery score.
